# ﻿*Polystichum oligodontum* (subg. Haplopolystichum, Dryopteridaceae), a new cave fern from Guangxi, China

**DOI:** 10.3897/phytokeys.266.156701

**Published:** 2025-11-25

**Authors:** You Nong, Ri-Hong Jiang, Chi Xiong, Qiu-Jun Wei, Gui-Yuan Wei, Xing-Yun Ji, Ke-Dao Lai, Hong-Jin Wei

**Affiliations:** 1 Guangxi Key Laboratory of Traditional Chinese Medicine Quality Standards; Guangxi Institute of Chinese Medicine & Pharmaceutical Science, No. 20–1 Dongge Road, Nanning, Guangxi, China Guangxi Key Laboratory of Traditional Chinese Medicine Quality Standards; Guangxi Institute of Chinese Medicine & Pharmaceutical Science Nanning China; 2 State Key Laboratory of Dao-di Herbs, Beijing 100700, China State Key Laboratory of Dao-di Herbs Beijing China; 3 Guangxi Key Laboratory of Special Non-wood Forest Cultivation and Utilization, Guangxi Forestry Research Institute, Nanning, 530002, China Guangxi Key Laboratory of Special Non-wood Forest Cultivation and Utilization, Guangxi Forestry Research Institute Nanning China; 4 Guangxi Key Laboratory of Plant Conservation and Restoration Ecology in Karst Terrain, Guangxi Institute of Botany, Guangxi Zhuang Autonomous Region and Chinese Academy of Sciences, Guilin, 541006, Guangxi, China Guangxi Zhuang Autonomous Region and Chinese Academy of Sciences Guilin China; 5 Eastern China Conservation Centre for Wild Endangered Plant Resources, Shanghai Chenshan Botanical Garden, Shanghai 201602, China Eastern China Conservation Centre for Wild Endangered Plant Resources, Shanghai Chenshan Botanical Garden Shanghai China

**Keywords:** Cave ferns, coarse tooth, karst area, serration pattern

## Abstract

*Polystichum oligodontum* (Dryopteridaceae), a new fern species in P. subg. Haplopolystichum from three karst caves in northwest Guangxi, China, is described and illustrated. This species is unique in having pinnae with a shallow or nearly free lobe on the acroscopic base and coarsely toothed distal margins. It resembles *P. membranifolium* and *P. kwangtungense* in its reflexed and oblong pinnae with an obtuse or rounded apex, but can be easily distinguished by its remote pinnae bearing a lobe at the acroscopic base. The new species also somewhat resembles *P. leveillei* in the nearly free basal lobe, but differs by its toothed upper pinna margins. Molecular phylogenetic analysis of 56 *Polystichum* species, based on the *rbc*L gene, supports the placement of *P. oligodontum* within P. sect. Sphaenopolystichum, with *P. alcicorne* as its closest relative. According to the IUCN Red List Categories and Criteria, the new species is assigned to the Critically Endangered (CR) category.

## ﻿Introduction

The fern genus *Polystichum* Roth is one of the largest and most taxonomically complex fern genera, comprising 180–230 (Kramer and Green 1990) or as many as 300 (Kung et al. 2001) species. There are 208 species (139 endemic) in two subgenera in China (Zhang et al. 2013) and more new species have been discovered in recent years (e.g. Lu et al. (2023); Wang et al. (2023); Xiong et al. (2023)). The genus is common in montane regions of both subtropical and temperate zones; many species occur on limestone substrates (Kung et al. 2001). The genus in China was revised in 2001 and included 13 sections, based on morphological characters — presence/absence of proliferous bulbils on the rachis, laminar dissection, shape and colour of the scales, serration form, texture and characteristics of the indusium (Kung et al. 2001). Recent chloroplast DNA sequence analysis has identified a strongly supported so-called BCPC (Lu et al. 2007) or CCPC clade (Li et al. 2008) containing species of Polystichum sect. Haplopolystichum s.l. (Zhang and He 2009), *Cyrtomidictyum* Ching, *Cyrtogonellum* Ching and Cyrtomium C. Presl subser. Balansana Ching & Shing (Little and Barrington 2003; Driscoll and Barrington 2007; Lu et al. 2007; Li et al. 2008). Recently, the Chinese *Polystichum* species were classified into two subgenera and 23 sections (Zhang et al. 2013; Péchon et al. 2016).

During our field surveys in a large karst region straddling the border between Bama and Fengshan Counties, Guangxi Zhuang Autonomous Region in the autumn of 2024, we found a unique *Polystichum* plant in three caves. After consulting relevant literature (e.g. Han et al. (2016, 2018); Zhang et al. (2019)) and checking relevant specimens, we confirmed that the unusual plant is new to science and is described below.

## ﻿Materials and methods

### ﻿Morphological analyses

The newly-discovered species was observed during the fieldwork. Its characters of stem, scales, frond, stipe, lamina, pinnae, sori and spores were studied. Voucher specimens were deposited at GXMI, IBK and CSH. For comparative morphology, we examined other *Polystichum* species using online images from the Chinese Virtual Herbarium (CVH, https://www.cvh.ac.cn/), Kew Herbarium Catalogue (http://apps.kew.org/herbcat/gotoHomePage.do) and JSTOR Global Plants (http://plants.jstor.org/). Morphological characters that distinguish it from all other species in the genus *Polystichum* are detailed.

Morphological features for the comparative species are mainly based on Zhang et al. (2013) and Han et al. (2016). Descriptions of the newly-discovered species are based on herbarium specimens. All measurements were made with a tape measure and callipers. The structure of the indumentum and its distribution were observed under a dissecting microscope at magnifications of more than 20× and then described. The ornamentation of spores was visualised using a scanning electron microscope (SEM). The untreated spores were fixed on aluminium stubs, coated with gold about 15 nm by iron sputtering (Hitachi E–101) and then examined using SEM (Hitachi S2400) at 7 kV. Magnification (600–1100×) was used for the whole spores and 6500x for the details.

### ﻿Genomic DNA extraction and sequencing

Total genomic DNA was extracted from silica-dried leaves using a modified CTAB method (Chen et al. 2014). The DNA samples were sent to Majorbio (http://www.majorbio.com/) for library construction and next-generation sequencing. A paired-end library with an insert size of 350 bp was constructed and sequencing was performed using the Illumina HiSeq 4000 platform. Approximately 1 Gb of raw reads was generated and subsequently filtered using the FASTX-Toolkit to remove adapter and low-quality reads (http://hannonlab.cshl.edu/fastx_toolkit/download.html). Complete chloroplast genome data was assembled using GetOrganelle v.1.7.7.0 (Jin et al. 2020).

### ﻿Phylogenetic analyses

To determine the phylogenetic placement of this species, we extracted the *rbc*L gene from complete plastid genome sequences of the new species. Accessions of the new species (GenBank Acc. No. PX215519, PX215520) were sequenced for this study. Additional sequences from *Polystichum* and related taxa were downloaded from GenBank, based on previous studies (Qiu et al. 2023; Chen et al. 2025) (see Suppl. material 1). The final dataset included 69 accessions representing 57 taxa, with 56 taxa belonging to *Polystichum* as the ingroup and *Cyrtomium macrophyllum* (Makino) Tagawa was selected as the outgroup.

All sequences were aligned using MAFFT within PhyloSuite v.1.2.3 (Katoh and Standley 2013; Zhang et al. 2020). The best substitution model (K2P+G4) was selected using ModelFinder (Kalyaanamoorthy et al. 2017), with the corrected Akaike Information Criterion (AICc). Bayesian Inference (BI) analysis was performed in MrBayes within PhyloSuite v.1.2.3 (Ronquist et al. 2012; Zhang et al. 2020). The Markov chains were run for 1,000,000 generations, with sampling every 1,000 generations and discarding the first 25% as burn-in. Maximum Likelihood (ML) analysis was conducted using IQ-TREE with 1,000 bootstrap replicates in PhyloSuite v.1.2.3 (Guindon et al. 2010; Minh et al. 2013; Nguyen et al. 2015; Zhang et al. 2020).

## ﻿Results and discussion

**Figure 1.**
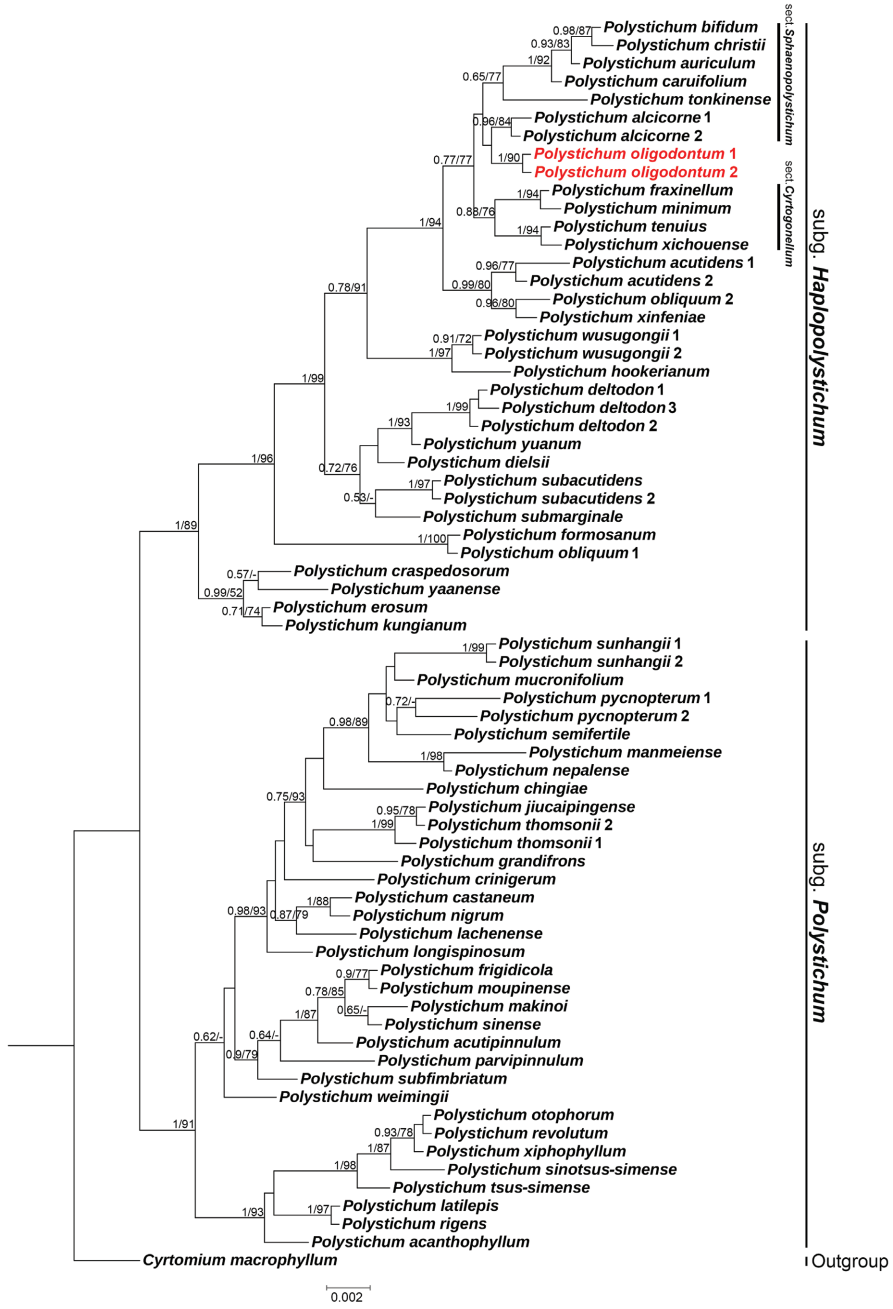
Bayesian tree of 56 species of *Polystichum*, based on the *rbc*L gene. The posterior probabilities (PP) of BI and bootstrap values (BS) of ML are listed at each node. The new species is highlighted in red.

The aligned *rbc*L sequences had a total length of 1,226 bp. The resulting phylogenetic tree was largely congruent with previous studies (Lu et al. 2007; Li et al. 2008; Péchon et al. 2016; Chen et al. 2025). The new species was situated within a clade that includes certain species from P. sect. Sphaenopolystichum Ching ex W.M.Chu & Z.R.He (such as *P. auriculum*, *P. bifidum*, *P. caruifolium*, *P. christii* and *P. tonkinense*), as well as sect. Cyrtogonellum (Ching) Li Bing Zhang (such as *P. fraxinellum*, *P. minimum*, *P. tenuius* and *P. xichouense*) (Fig. 1).

**Figure 2.**
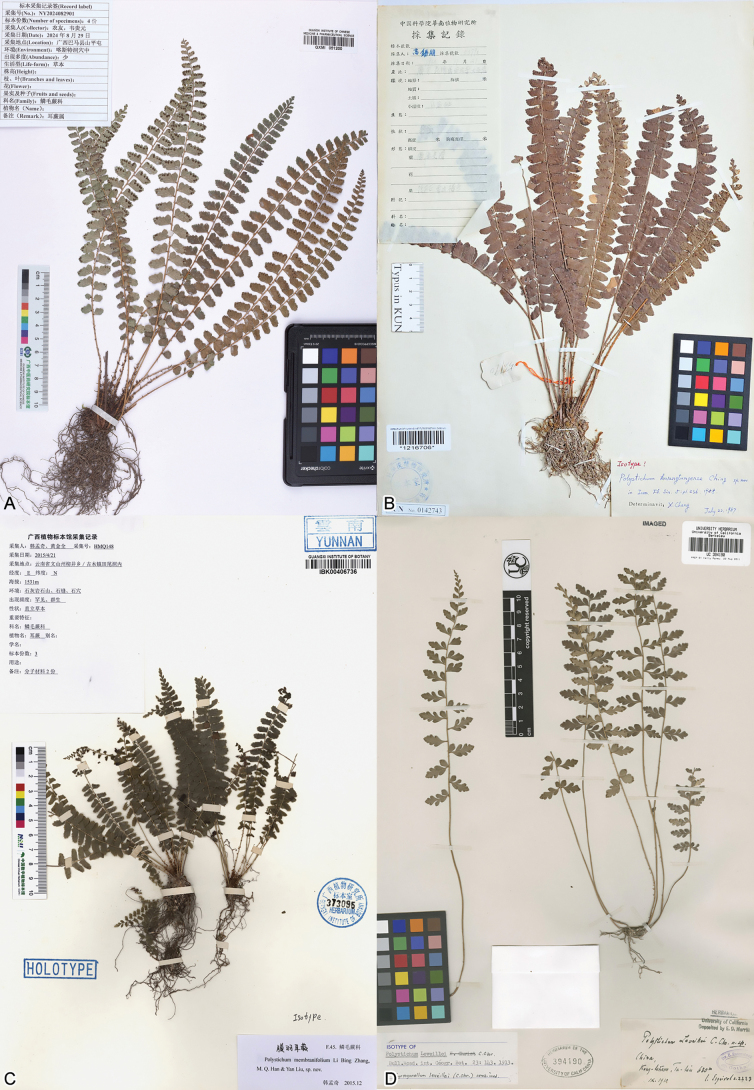
Type specimens of the new taxon and three morphologically related species; **A.***Polystichum oligodontum*; **B.***P. kwangtungense*; **C.***P. membranifolium*; **D.***P. leveillei*.

*Polystichum oligodontum* is characterised by pinnae that display the following features: (1) somewhat reflexed, (2) oblong, (3) an acroscopic basal lobe, (4) rounded teeth and (5) an obtuse or rounded apex. Three species, *P. membranifolium* Li Bing Zhang, M.Q. Han & Yan Liu (sharing 1, 2, 4 and 5), *P. kwangtungense* Ching (sharing 1, 2 and 5) and *P. leveillei* C.Chr. (sharing 2, 3, 4 and 5), were selected for morphological comparison with *P. oligodontum*, as shown in Fig. 2 and Table 1. A few other species, such as *P. dielsii* Christ and *P. pseudodeltodon* Tagawa, also share the features 2 and 5, but their pinnae are not reflexed. *Polystichum oligodontum* and *P. membranifolium* exhibit the greatest similarity in their rhizome scale shape, lamina shape, pinna and sorus number, pinna orientation and tooth form. However, *P. oligodontum* differs by its larger stature, papery frond texture, submarginal sori and pinnae sometimes with a partially separated lobe at the acroscopic base (Fig. 2C). *Polystichum oligodontum* shares a few similarities with *P. kwangtungense*, but it can be easily distinguished by its smaller stature (9.6–30 cm vs. 30–42 cm), attenuate lamina base (vs. lamina base nearly as wide as middle part), fewer pinnae (13–22 pairs vs. 30–35 pairs) that are remote (vs. contiguous or imbricate), pinnae division (with a lobe on acroscopic side vs. slightly acute-auriculate on acroscopic side) and fewer sori (1–6 above mid-rib vs. up to 9 above mid-rib) (Fig. 2B). *Polystichum oligodontum* is somewhat similar to *P. leveillei* by having a lobe at the acroscopic pinna base, papery frond and submarginal sori. However, the latter has many characters distinct from those of *P. oligodontum*, such as lanceolate and subulate rhizome scales and 8–12 pairs of ascendent, remote pinnae reaching 45 × 16 mm. In addition, *P. leveillei* has irregularly lobed pinnae, usually with a free lobe at the acroscopic pinna base (Fig. 2D). These features are distinct from those of other species within the sect. Haplopolystichum.

**Table 1.** Main morphological differences amongst *Polystichum oligodontum*, *P. membranifolium*, *P. leveillei* and *P. kwangtungense*.

Morphological traits	*P. oligodontum*	*P. membranifolium*	*P. leveillei*	*P. kwangtungense*
**Rhizome scales**	ovate to oblong, denticulate at margins	ovate to ovate-lanceolate, nearly entire at margins	lanceolate and subulate, irregularly ciliate at margins	ovate-lanceolate, with shortly filiform teeth at margins
**Fronds**	10–30 cm long	12–17 cm long	9–35 cm long	30–42 cm long
**Stipe**	2–8.5 cm long	3–5 cm long	6–16 cm long	10–12 cm long
**Lamina**	lanceolate, oblong-lanceolate or oblanceolate, 7.5–30 × 1.8–3 cm	oblanceolate, 7–13 × 0.7–2.1 cm	narrowly elliptic-lanceolate, 7–22 × 3–8 cm	nearly linear-lanceolate, 20–30 × ca. 2.5 cm
**Rachis scales**	lanceolate, sparsely denticulate at margins	subulate to ovate-lanceolate, regularly ciliate at margins	lanceolate and subulate, irregularly ciliate at margins	ovate-lanceolate, with shortly filiform teeth at margins
**Pinnae**	13–22 pairs, 8.5–15 × 4.5–8 mm, closely spaced, oblong, reflexed, obtuse at apex	20–26 pairs, 5–9 × 2–4 mm, contiguous, oblong, reflexed, rounded at apex	8–12 pairs, 26–45 × 10–16 mm, remote, oblong, ascendant, obtuse at apex	30–35 pairs, 10–14 × 6–7 mm, contiguous or imbricate, oblong, reflexed, obtuse at apex
**Acroscopic pinna margin**	toothed, base with a broadly deltoid auricle or sometimes lobed to half or more of the pinna	toothed, base somewhat articulate	irregularly lobed, base with a free lobe	shallowly repand or shallowly toothed, base slightly acute-auriculate, distal
**Pinna teeth**	rounded and with short spinules	rounded and with short spinules	rounded but without spinules	with short and fine acute tip
**Frond texture**	papery	membranous	papery	papery
**Sori**	close to pinna margin, 1–6 above mid-rib, 0–3 below mid-rib	slightly close to pinna margin, 1–6 above mid-rib, 0–1 below mid-rib	close to pinna margin	relatively close to pinna margin, up to 9 above mid-rib, 1–3 below mid-rib on distal part of pinnae, sometimes missing below mid-rib
**Indusia**	margin repand or slightly erose, evanescent	margin entire, evanescent	margin slightly erose	margin entire, falling off when mature

### ﻿Taxonomy

#### ﻿ Polystichum oligodontum

Taxon classificationPlantaePolypodialesDryopteridaceae

Y.Nong, R.H.Jiang & C.Xiong sp. nov.

00D2F038-4148-52B6-AE8C-A6E01E24C429

urn:lsid:ipni.org:names:77372481-1

Figs 3, 4, 5

##### Type.

**China**. • Guangxi: Bama County, Jiazhuan Town, Renxiang Village, 24°19'36"N, 107°6'19"E, in a limestone cave, elev. 533 m, 29 August 2024, *You Nong NY2024082901* (holotype: GXMI051200!; isotypes: CSH!, GXMI!, IBK!).

##### Diagnosis.

*Polystichum oligodontum* is unique by the pinna acroscopic side with a shallow or nearly free lobe at the base and coarsely toothed along distal margins.

**Figure 3.**
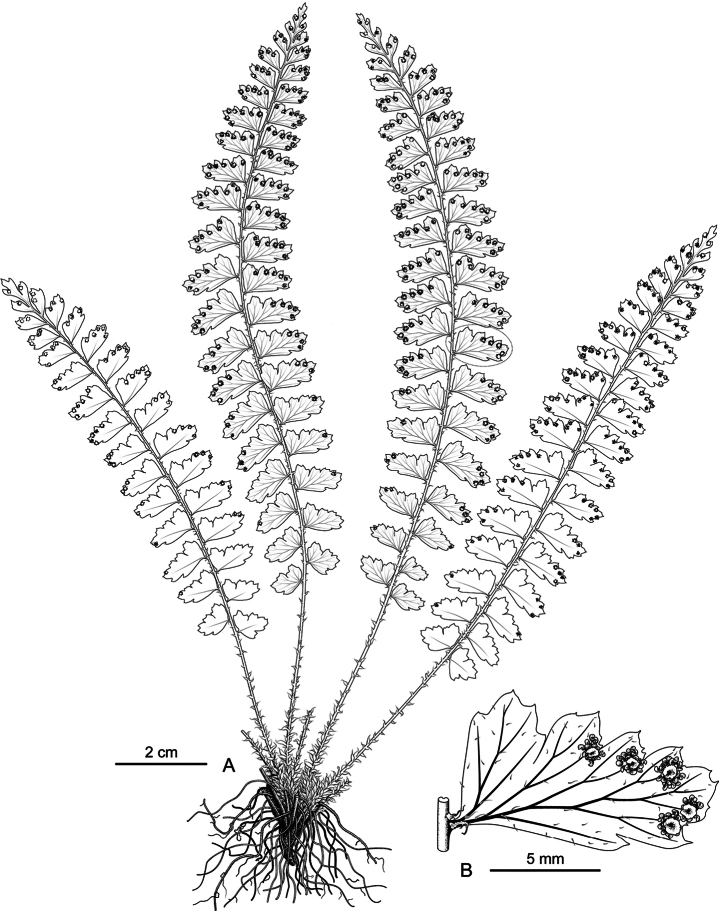
*Polystichum oligodontum* (based on paratypeDXH240718-28 in CSH). **A.** Habit; **B.** Portion of rachis with pinna. Illustrated by Hong-Jin Wei.

**Figure 4.**
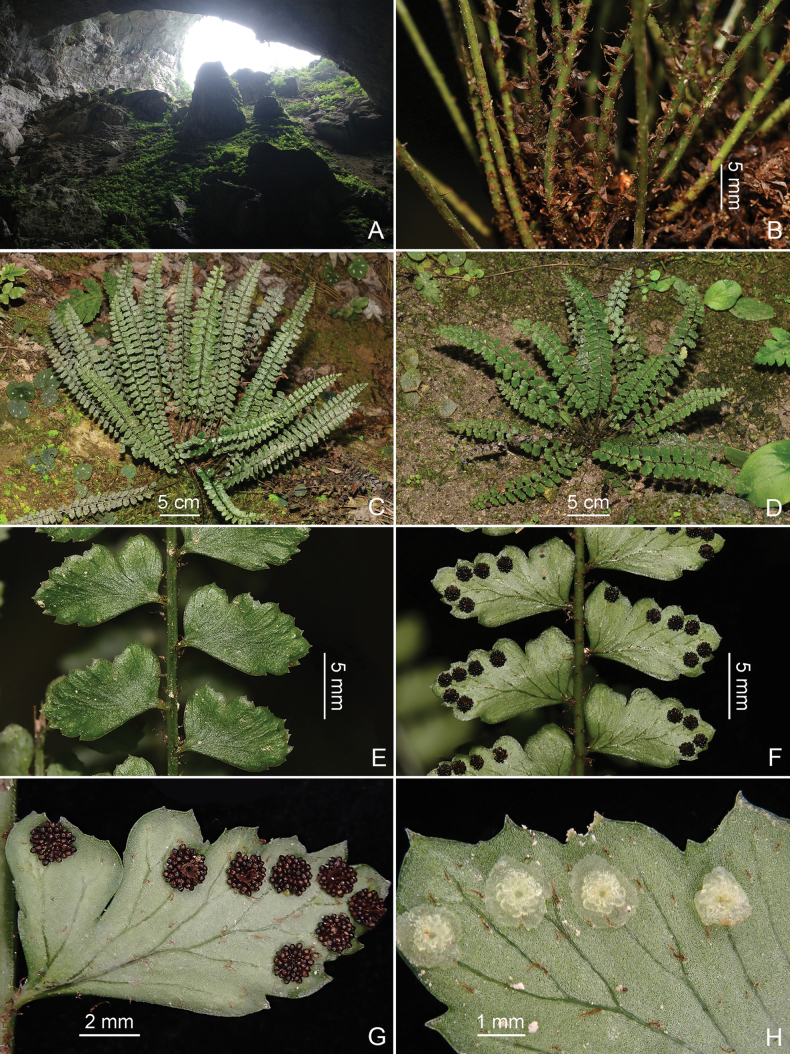
*Polystichum oligodontum* (from Bama county). **A.** Inside view of the cave where the new species was discovered; **B.** Lower portions of stipes showing scales; **C, D.** Habits; **E.** Portions of laminae (adaxially); **F.** Portions of laminae (abaxially); **G.** Pinna showing sori; **H.** Portion of pinna showing indusia.

##### Description.

**Plants** perennial, evergreen. **Rhizome** short, erect, densely scaly. **Fronds** caespitose, 6–20 per rhizome, (6.5–)10–19(–30) cm long; **stipe** pale green when dry, (1.5–)2–5(–8.5) cm long, 0.3–0.5(–1) mm in diam. near middle, shallowly grooved adaxially, base densely covered with scales, upward scales sparse; **scales** ovate to oblong, brown to dark brown, apically attenuate to short-caudate, membranous, 2.5–4 × ca. 1.2 mm, spreading, denticulate on margin. **Lamina** 1-pinnate, lanceolate, oblong-lanceolate or oblanceolate, widest at or above middle, (5–)7.5–14(–23) × (1.4–)1.8–2.5(–3) cm, slightly narrower towards base, base 0.8–1.7 cm wide, apex acute to short-acuminate; rachis pale green, shallowly grooved adaxially, sparsely covered with loose scales abaxially, scales lanceolate, brown, denticulate on margin. **Pinnae** 13–22 pairs, usually alternate, occasionally basal 1–2 pairs opposite, remote or proximate, short-stalked, stalks ca. 0.5 mm, abaxially with 1–2 scales similar to those on rachis, upper pinnae spreading to slightly ascending, pinnae below middle usually reflexed; lowest 2–4 pairs of pinnae 2–10(–14) mm apart; lowest pair sub-quadrangular, obliquely ovate or broadly flabellate, 0.8–5(–7) × 0.4–3(–5) mm; middle pinnae (6.3–)8.5–12(–15) × (3.5–)4.5–6(–8) mm, oblong, apex obtuse, subobtuse or subacute, with 1 or 2 broad, slightly mucronate teeth, base asymmetrical, acroscopic base wider, somewhat auriculate, auricles partially separated by a deep or shallow sinus, sometimes forming a nearly free lobe, with 4–5 acute, slightly mucronate teeth along upper margin, proximal margins of auricles slightly curved or straight, parallel or subparallel to rachis; upper acroscopic margin of pinnae nearly straight, coarsely toothed, with 2–3 increasingly shallow sinuses between teeth distally, teeth subacute to rounded-apiculate; basiscopic base of pinnae narrowly cuneate; basiscopic margins entire below apex, straight or nearly so, mostly acutely angled to rachis below; distal basiscopic margin curved upwards; adaxial surface glabrous, abaxial surface sparsely covered with lanceolate microscales, scales ca. 0.6–1 mm long, greyish-brown, denticulate on margin. **Frond texture** papery when dried, green adaxially; venation free, visible abaxially, indistinct adaxially, lateral veins pinnate from base upwards, dichotomous or simple above mid-rib, simple or dichotomous below mid-rib, extending into teeth, but not reaching pinna margin. **Sori** ca. 1 mm in diam., near pinna margins, terminal on shorter veinlets, 1–6 above mid-rib, often 0–3 below mid-rib, separate or confluent when mature. **Indusia** small, greyish-brown, membranous, evanescent, margin repand or slightly erose.

**Figure 5.**
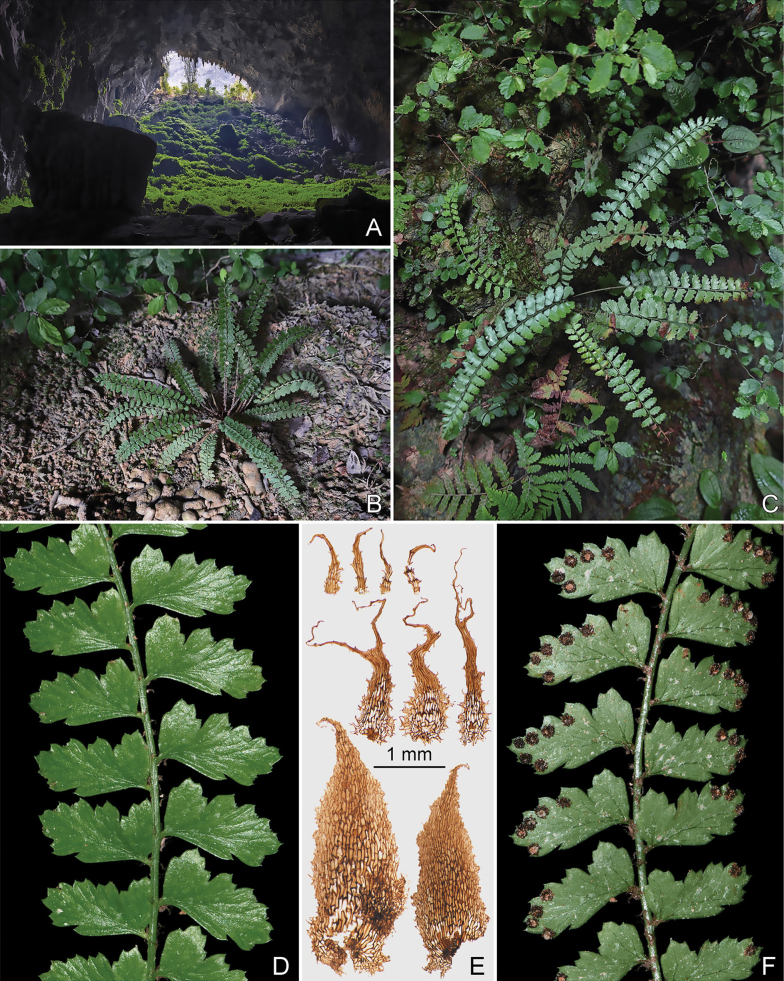
*Polystichum oligodontum* (from Fengshan County). **A.** Inside view of the cave where the new species was discovered; **B, C.** Habit; **D.** Portions of laminae (adaxially); **E.** Scales from stipes base (bottom), pinna base (middle) and pinna surface (top); **F.** Portions of lamina (abaxially).

##### Spore morphology.

**Figure 6.**
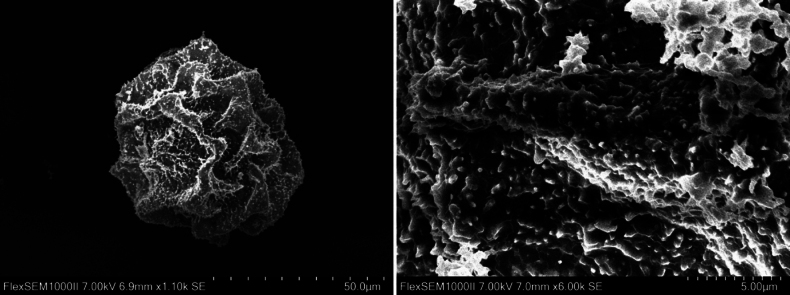
Scanning electron micrographs of spore of *Polystichum oligodontum* (from type specimen GXMI051200). Left image depicting proximal view of spore, while right image showing distal view of spore surface.

Perispore with coarse, irregularly inflated, partially interconnected folds and both cristate and echinulate microstructure, the latter on the spore surface and on the cirstae (Fig. 6).

##### Geographical distribution.

**Figure 7.**
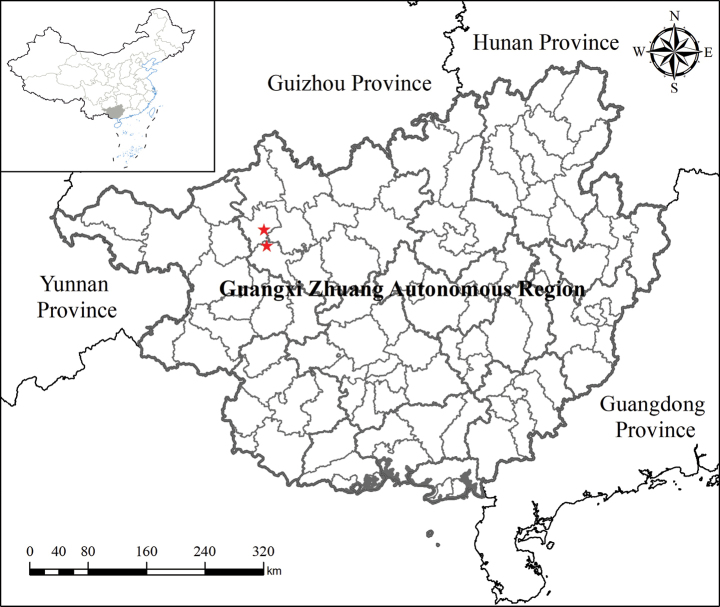
Distributions of *Polystichum oligodontum* (red stars) in Guangxi, China.

*Polystichum oligodontum* is found so far only in three limestone caves in Guangxi Zhuang Autonomous Region, south China. Two of them are located in Xinglong Village, Fengcheng Town, Fengshan County and one of them in Renxiang Village, Jiazhuan Town, Bama County (Fig. 7).

##### Ecology.

*Polystichum oligodontum* was discovered in karst caves at an elevation of 530–770 m, growing on limestone rocks with sizes less than 0.1 m^3^ or on ground covered with thin sandy soil, at a distance of about 50–100 m to the cavern mouths.

##### IUCN Red List category.

Three populations with approximately 40 individuals were found respectively at three caves located in a karst region without any protection. The distance between the two closest caves is about 150 m. The third cave is about 17 km away from them. There are many villages in this region. The closest village is about 400 m (in a straight line) away from the cave, the type locality. However, this cave is easily visited and disturbed by the nearby villagers and some domestic animals, such as cattle and goats. The new species could disappear at any time if the habitat was seriously damaged. The status of the new species should be CR – Critically Endangered category, based on current information and following IUCN (the International Union for Conservation of Nature and Natural Resources) guidelines (IUCN 2022).

##### Etymology.

The specific epithet is derived from the Latin, *oligodontum*, meaning “few teeth”, and referring to the number of the teeth on pinna margins.

##### Chinese name.

稀齿耳蕨 (xī chǐ ěr jué).

##### Paratypes.

**China** • Guangxi: Fengshan County, Fengcheng Town, Xinglong Village, in a karst cave, elev. 769 m, 18 July 2024, *Chi Xiong & Xiao-Ying Fu DXH240718-28* (IBK!, CSH!); • ibid., elev. 736 m, 22 March 2025, *Chi Xiong DXGJ250322-52* (CSH!) & *DXGJ250322-54* (CSH!).

##### Taxonomic notes.

*Polystichum oligodontum* has 1-pinnate lamina and lanceolate stipe and rachis scales and is clearly a member of sect. Haplopolystichum within P. subg. Haplopolystichum. Morphologically, *P. oligodontum* can be readily distinguished from any species in the section in terms of pinna serration pattern, namely, the pinnae proximally exhibit a broad and 2–4 (or more) toothed auriculate lobe which is sometimes partially separated by a deep or shallow incision, distally followed with some coarse teeth along the acroscopic margin. This pinna serration pattern can be shown from some precocious *Polystichum* species such as *P. normale* Ching ex P.S.Wang & Li Bing Zhang in sect. Crucifilix Tagawa and *P. bifidum* Ching and *P. pseudolanceolatum* Ching ex P.S. Wang in sect. Sphaenopolystichum Ching ex W.M.Chu & Z.R.He. However, the new species can be easily recognised by its pinnae in having fewer teeth and shallower incisions on pinna margin, as well as shorter size.

## Supplementary Material

XML Treatment for Polystichum oligodontum

## ﻿Additional information

### Use of AI

No use of AI was reported.

### Author ORCIDs

You Nong https://orcid.org/0000-0001-7004-0946

Ri-Hong Jiang https://orcid.org/0000-0003-4908-3487

Chi Xiong https://orcid.org/0000-0002-9082-2539

Qiu-Jun Wei https://orcid.org/0009-0002-5799-6323

Gui-Yuan Wei https://orcid.org/0000-0003-0652-1213

Xing-Yun Ji https://orcid.org/0009-0002-4907-6850

Ke-Dao Lai https://orcid.org/0000-0003-0807-6344

Hong-Jin Wei https://orcid.org/0000-0002-3679-6683

### Data availability

All of the data that support the findings of this study are available in the main text.

## Supplementary materials

Supplementary material 1 Voucher information for phylogenetic analyses and GenBank accession numbers Data type docx https://binary.pensoft.net/file/1477524This dataset is made available under the Open Database License (http://opendatacommons.org/licenses/odbl/1.0/). The Open Database License (ODbL) is a license agreement intended to allow users to freely share, modify, and use this Dataset while maintaining this same freedom for others, provided that the original source and author(s) are credited.You Nong, Ri-Hong Jiang, Chi Xiong, Qiu-Jun Wei, Gui-Yuan Wei, Xing-Yun Ji, Ke-Dao Lai, Hong-Jin Wei
