# Supplementary material for: ﻿Polystichum oligodontum (subg. Haplopolystichum, Dryopteridaceae), a new cave fern from Guangxi, China
Source: PhytoKeys. 2025 Nov 25;266:297–310. doi: 10.3897/phytokeys.266.156701 (PMC12673335; doi:10.3897/phytokeys.266.156701)
Supplement: Supplementary material 1 — Voucher information for phylogenetic analyses and GenBank accession numbers [file phytokeys-266-297_article-156701__-s001.docx]

**Suppl. material 1.** Voucher information for phylogenetic analyses and GenBank accession numbers.

| **Taxon** | **Voucher** | **Locality** | ***rbc*L** |
| --- | --- | --- | --- |
| *Cyrtomium macrophyllum* | L.-B. Zhang et al. 5771 (CDBI, CTC, MO) | Guizhou, China | KU244740 |
| *Polystichum acanthophyllum* | Li Bing Zhang and Hai He 64 (MO) | Yunnan, China | KU244712 |
| *Polystichum acutidens* 1 | ZXC001588 (TAIF) | Taiwan, China | PP560723 |
| *Polystichum acutidens* 2 | Wade5068 (TAIF) | Lam Dong, Vietnam | PP560113 |
| *Polystichum acutipinnulum* | Li Bing Zhang and Hai He 5401 (CDBI, CTC, MO) | Guangxi, China | KU244732 |
| *Polystichum alcicorne* 1 | Hai He 919 (CTC) | Chongqing, China | KU244759 |
| *Polystichum alcicorne* 2 | L.-B. Zhang et al. 816 (CDBI, PYU) | Guizhou, China | KU244723 |
| *Polystichum auriculum* | Li Bing Zhang and Hai He 5014 (CDBI, CTC) | Yunnan, China | KU244758 |
| *Polystichum bifidum* | Li Bing Zhang and Hai He 5012 (CDBI, CTC) | Yunnan, China | KU244757 |
| *Polystichum caruifolium* | Li Bing Zhang and Cehong Li 4795 (CDBI, MO) | Sichuan, China | KU244724 |
| *Polystichum castaneum* | Hai He HG091 (CTC) | Sichuan, China | KU244730 |
| *Polystichum chingiae* | L.-B. Zhang & H. He 4921 (CDBI, MO) | Yunnan, China | KU244720 |
| *Polystichum christii* | SG Lu/H13 (PYU) | China | AY545486 |
| *Polystichum craspedosorum* | Zhangming Zhu 568 (CDBI) | Henan, China | KU244761 |
| *Polystichum crinigerum* | L.-B. Zhang & H. He 4847 (CDBI, CTC, MO) | Yunnan, China | KU244722 |
| *Polystichum deltodon* 1 | Li Bing Zhang and Hai He 94 (CTC, MO) | Yunnan, China | KU244710 |
| *Polystichum deltodon* 2 | L.-B. Zhang et al. 4749 (CDBI, MO) | Sichuan, China | KU244703 |
| *Polystichum deltodon* 3 | He Hai Polystichum_nigrum_HG066 (CTC) | Chongqing, China | KU244726 |
| *Polystichum dielsii* | L.-B. Zhang & H. He 447 (CDBI, CTC, MO) | Guizhou, China | KU244709 |
| *Polystichum erosum* | L.-B. Zhang et al. 4775 (CDBI) | Sichuan, China | KU244763 |
| *Polystichum formosanum* | T. Ranker 2073(COLO) | Taiwan, China | EF177337 |
| *Polystichum fraxinellum* | LJM 002 (KUN) | Yunnan, China | AY694810 |
| *Polystichum frigidicola* | Hai He HG071 (CTC) | Sichuan, China | KU244729 |
| *Polystichum grandifrons* | L.-B. Zhang & H. He 5025 (CDBI, CTC, MO) | Yunnan, China | KU244719 |
| *Polystichum hookerianum* | L.-B. Zhang & H. He 5394 (CDBI, CTC, MO) | Guangxi, China | KU244738 |
| *Polystichum jiucaipingense* | Li Bing Zhang and Hai He 700 (CDBI, CTC, MO) | Guizhou, China | KU244702 |
| *Polystichum kungianum* | Hai He and Yongqing Yang 016 (CTC) | Chongqing, China | KU244764 |
| *Polystichum lachenense* | Fay-Wei Li 1403 (MO) | Taiwan, China | KU244743 |
| *Polystichum latilepis* | Liang Zhang and Zhangming Zhu 1083 (CDBI) | Hubei, China | KU244735 |
| *Polystichum longispinosum* | L.-B. Zhang & C.-H. Li 4786 (CDBI, MO) | Sichuan, China | KU244706 |
| *Polystichum makinoi* | Lu/C61 (PYU) | Yunnan, China | AY545494 |
| *Polystichum manmiense* | Li Bing Zhang and Hai He 4911 (CDBI, CTC, MO) | Yunnan, China | KU244717 |
| *Polystichum minimum* | LJM 047 (KUN) | Chongqing, China | AY694812 |
| *Polystichum moupinense* | Hai He HG118 (CTC) | Sichuan, China | KU244731 |
| *Polystichum mucronifolium* | L.-B. Zhang & H. He 4912 (CDBI, CTC, MO) | Yunnan, China | KU244715 |
| *Polystichum nepalense* | L.-B. Zhang & H. He 4928 (CDBI) | Yunnan, China | KU244713 |
| *Polystichum nigrum* | Hai He HG066 (CTC) | Sichuan, China | KU244728 |
| *Polystichum obliquum* 1 | ZXC001436 (TAIF) | Taiwan, China | PP560710 |
| *Polystichum obliquum* 2 | X.-F. Gao et al. T05-409 (KUN) | Gandaki, Nepal | OR543846 |
| *Polystichum oligodontum* 1 | Chi Xiong DXH250612-18(IBK, CSH) | Bama, Guangxi, China | PX215520 |
| *Polystichum oligodontum* 2 | Chi Xiong DXH240718-28(IBK, CSH) | Fengshan, Guangxi, China | PX215519 |
| *Polystichum otophorum* | Li Bing Zhang and Hai He 6 (CTC, MO) | Sichuan, China | KU244711 |
| *Polystichum parvipinnulum* | s.c. 22959 (TNS) | Taiwan, China | KU244739 |
| *Polystichum pycnopterum* 1 | L.-B. Zhang & H. He 4938 (CDBI, CTC, MO) | Yunnan, China | KU244714 |
| *Polystichum pycnopterum* 2 | S.-G. Lu/B11 (PYU) | Yunnan, China | AY545502 |
| *Polystichum revolutum* | Li Bing Zhang and Cehong Li 4773 (CDBI, CTC, MO) | Sichuan, China | KU244708 |
| *Polystichum rigens* | Liang Zhang and Zhangming Zhu 1082 (CDBI) | Hubei, China | KU244736 |
| *Polystichum semifertile* | L.-B. Zhang & H. He 4903 (CDBI, CTC, MO) | Yunnan, China | KU244718 |
| *Polystichum sinense* | Le Péchon 1225 (CDBI, REU) | Réunion, France | KU244755 |
| *Polystichum sinotsus-simense* | Li Bing Zhang and Hai He 5690 (CDBI, CTC, MO) | Guangxi, China | KU244733 |
| *Polystichum subacutidens* 1 | Lu SG/D8 (PYU) | Yunnan, China | AY545488 |
| *Polystichum subacutidens* 2 | J.-M. Lu 060 (KUN) | China | DQ508787 |
| *Polystichum subfimbriatum* | L.-B. Zhang & H. He 4837 (CDBI, CTC, MO) | Yunnan, China | KU244721 |
| *Polystichum submarginale* | L.-B. Zhang & C.-H. Li 4793 (CDBI, MO) | Sichuan, China | KU244707 |
| *Polystichum sunhangii* 1 | Liang Zhang et al. 4582 (KUN) | Xizang, China | OR543843 |
| *Polystichum sunhangii* 2 | Liang Zhang et al. 4548 (KUN) | Xizang, China | OR543838 |
| *Polystichum tenuius* | Liang Zhang 1160 (CDBI) | Chongqing, China | KU244705 |
| *Polystichum thomsonii* 1 | Fay-Wei Li 1391 (MO) | Taiwan, China | KU244744 |
| *Polystichum thomsonii* 2 | L.-B. Zhang & H. He 4824 (CDBI, CTC, MO) | Yunnan, China | KU244716 |
| *Polystichum tonkinense* | Li-Bing Zhang et al. 473 (CDBI, CTC, MO) | Guizhou, China | KU244760 |
| *Polystichum tsus-simense* | Hai He 1024 (CTC) | Sichuan, China | KU244734 |
| *Polystichum weimingii* | Li Bing Zhang et al. 5985 (CDBI, CTC, MO) | Yunnan, China | KU253818 |
| *Polystichum wusugongii* 1 | Liang Zhang et al. 4219 (KUN) | Xizang, China | OR543844 |
| *Polystichum wusugongii* 2 | Liang Zhang et al. 4709 (KUN) | Xizang, China | OR543842 |
| *Polystichum xichouense* | SG Lu/J30 (PYU) | Yunnan, China | DQ054515 |
| *Polystichum xinfeniae* | L.Zhang et al. 4538 (KUN) | Xizang, China | OR543840 |
| *Polystichum xiphophyllum* | Li Bing Zhang et al. 636 (CDBI, CTC, MO) | Guizhou, China | KU244701 |
| *Polystichum yaanense* | L.-B. Zhang et al. 4745 (CDBI) | Sichuan, China | KU244762 |
| *Polystichum yuanum* | W. M. Zhu and Z. R. He s.n. (CDBI, PYU) | Yunnan, China | KU244737 |
